# Evaluation of a biocoagulant from devilfish invasive species for the removal of contaminants in ceramic industry wastewater

**DOI:** 10.1038/s41598-022-14242-6

**Published:** 2022-06-15

**Authors:** Miguel Mauricio Aguilera Flores, Nahum Andrés Medellín Castillo, Verónica Ávila Vázquez, Raúl González García, Antonio Cardona Benavides, Candy Carranza Álvarez

**Affiliations:** 1 Multidisciplinary Graduate Program in Environmental Sciences, Autonomous University of San Luis Potosi, Av. Manuel Nava 201, Col. Zona Universitaria Poniente, 78210 San Luis Potosí, SLP Mexico; 2 Interdisciplinary Professional Unit of Engineering, Campus Zacatecas, Instituto Politécnico Nacional, Blvd. del Bote 202 Cerro del Gato Ejido la Escondida, Col. Ciudad Administrativa, 98160 Zacatecas, ZAC México; 3 Graduate Studies and Research Center, Faculty of Engineering, Autonomous University of San Luis Potosi, Av. Manuel Nava No. 8, Col. Zona Universitaria Poniente, 78290 San Luis Potosí, SLP México; 4 Graduate Studies and Research Center, Faculty of Chemical Sciences, Autonomous University of San Luis Potosi, Av. Manuel Nava No. 6, Col. Zona Universitaria Poniente, 78210 San Luis Potosí, SLP México; 5 Multidisciplinary Academic Unit, Huasteca Zone, Autonomous University of San Luis Potosi, Romualdo del Campo 501, Fracc. Rafael Curiel, 79060 Ciudad Valles, SLP México

**Keywords:** Chemical engineering, Environmental chemistry, Biotechnology, Environmental sciences

## Abstract

This study evaluated the effectiveness of a biocoagulant produced from the devilfish invasive species and its combination with two chemical coagulants (aluminum sulfate and ferric sulfate) to remove turbidity, chemical oxygen demand, and total suspended solids in ceramic industry wastewater using a combined experimental design of Mixture-Process. This design optimized the coagulation process and evaluated the effects and interactions between mixture components and coagulant doses. An analysis of variance was used to analyze the experimental data obtained in the study, and the response surface plots by response type (turbidity, chemical oxygen demand, and total suspended solids) were obtained. Results showed that the coagulation treatment could be technically and economically feasible since efficiencies of turbidity, chemical oxygen demand, and total suspended solids removal of 74, 79, and 94% could be achieved using an optimal coagulant dose of 800 mg/L with a mixture of 35% biocoagulant and 65% ferric sulfate. Analysis of variance results showed that the models are significant, and the lack of fit is not required according to the probability value (*p* value), which were < 0.0001, and > 0.05, respectively. Hence, the experimental data were fitted to a combined reduced special cubic x linear model. These results support the use of devilfish meal as a biocoagulant, being more feasible in dual systems when mixed with ferric sulfate.

## Introduction

The introduction of invasive species has been identified as the most significant cause of global change^1,2^ because it is the second most common cause of species extinction (after habitat destruction)^3^. This activity causes ecological impacts that can extend throughout the food chain, altering the functioning of the elements that make up an ecosystem and deteriorating ecosystem and environmental services throughout the world^1,4,5^. Similarly, it has been reported that these species have significant socioeconomic and health impacts that must be considered together with the environmental ones^6–8^.

The challenges for controlling and managing invasive species are approached from different points of view. One of these points is how to promote their management^9,10^, establishing policies that integrate the effects (cost–benefit) of the introduction, control, management, or eradication of these species on ecosystems. Therefore, one alternative is their sustainable use, considering causing their death to improve the control of biological invasions according to its type, providing some use or application to reduce their category as invasive species^2,11–13^.

Devilfish is an invasive species of the Loricariidae family native to South America of the Amazon basin, but it has been introduced in several countries, as in Mexico^14,15^. Devilfish has become an environmental problem in the country, at least in the last 20 years^16^. Its presence affects other fish species since they destroy their eggs and compete for food^17^. One of the most significant impacts is the negative impact that they generate on the economy and welfare of fishers who sell Nile tilapia since have reduced its population by 83%, generating a collapse of commercial fishing that has affected the state of health, unemployment, and the emigration of fishers^14,18^. Due to the environmental and socioeconomic impacts that this species generates, fishers cause their death to better control these biological invasions with the purpose of their well-being or economy is not harmed. However, when these species are killed, they are not put to any use and are only generated as waste^11^.

Devilfish biomass has been studied to produce adsorbent materials^19^ and bioanodes of microbial fuel cells^20,21^ as low-cost alternatives for water and wastewater treatment. Likewise, its meal has been studied as a partial replacement of commercial fish meal in fish feed formulations^22–24^ since it is a good source of proteins, minerals, and fats^25^. The collagen is the principal constituent of the intramuscular connective tissue of a fish, exerting a vital function in the texture of their meat^26^ and has been mainly used as the ideal coagulation material^27^. Even though the devilfish meat has a high-quality protein content to be consumed with nutritional benefits, it has been reported the possible health risks in the direct consumption of devilfish meat, or indirect to be used the devilfish meal as food for fishes due to exposure to heavy metals such as Cadmium, Chromium, Manganese, and Lead^16^. In this sense, one alternative to explore the use of the devilfish biomass is producing a biocoagulant applied in wastewater treatment.

Biocoagulants have been studied due to their capability to improve the removal efficiency of contaminants in the water^28^. These materials are based on natural polymers like cellulose, mucilage, and collagen, and they are produced from the animal base, plant base, microorganisms, and bacteria^28,29^. Biocoagulants used in wastewater treatment are mainly based on plant bases. Some of them are banana pith^30^, Jatropha curcas^31^, and Moringa oleifera^32^. The use of animal biomass has been scarcely reported for this purpose, but it attracts more attention when biocoagulants are produced from invasive species.

The ceramic industry wastewater requires a primary treatment based on coagulation/flocculation/sedimentation processes since it contains inorganic matter associated with sand and clays. Hanife^33^ reported the 95% of Chemical Oxygen Demand (COD) removal in the ceramic industry wastewater using a dose of 3.3 g/L of alum at pH 5. It was better than ferric chloride. However, it has been reported that chemical coagulants (iron salts and aluminum salts) have contributed to toxicological issues and environmental effects caused by the constant consumption and large doses during the treatment^28^. Therefore, this treatment based on natural materials, as the use of biocoagulants, has been a topic of interest in the investigation due to their positive effects when their use reduces or avoids the possible toxicity for human health and biota caused by using chemical agents in the wastewater treatment^28^. Biocoagulants produced from *Jatropha curcas*, *Moringa oleifera*, *Dolichos lablab*, and *Cicer arietinum* seeds have been proved in the treatment of synthetic water with kaolin and clay materials, reporting efficiencies of turbidity removal > 88%^31,34^.

In this context, this work aims to evaluate the effectiveness of a biocoagulant from the devilfish invasive species to remove contaminants of the ceramic industry wastewater, studying it at different concentrations and using only the biocoagulant or its combination with chemical coagulants (aluminum sulfate and ferric sulfate). This study is based on an experimental design that allowed to find the optimal operation conditions (optimal doses and proportions of mixture) to treat mentioned wastewater. This work presents an integrated solution to two problems, the first one the exploitation of the invasive species devilfish as biomass helpful in controlling its population in ecosystems that it does not belong and the second one the production of a biocoagulant that can be used as primary treatment in wastewaters, reducing the use de chemical agents.

## Material and methods

### Feedstock

This research produced a biocoagulant from devilfish meat, and its effectiveness was compared with two chemical agents used as conventional coagulants. The non-live devilfish samples were provided by the Fishermen's Society of Tenosique (Tabasco, Mexico). Four fishes weighing between 200 g and 1 kg (giving 1.7 kg) were used, obtaining approximately 0.5 kg of meat.

Ceramic industry wastewater was used as a case study to evaluate the effectiveness of the biocoagulant. The samples were collected at the wastewater discharge point of a ceramic industry (located on latitude 22°55′23″ N and longitude 102°40′40″ W). This wastewater was selected because the stages required for its treatment are at the primary level, using sedimentation-coagulation-flocculation processes since it contains inorganic matter associated with sand and clays.

The chemical agents used as conventional coagulants were aluminum sulfate and ferric sulfate (reagent grade, Mca. Meyer). Furthermore, a commercial flocculant (anionic polymer) was used for all the tests. This flocculant was prepared at 1 mg/L and was provided by a Mexican company.

### Preparation, thermogravimetric analysis, and heavy metal content of the biocoagulant

The biocoagulant was prepared from a devilfish meal. The devilfish samples of different sizes were pooled together. Their visceral organs were eliminated, and then they were cleaned and rinsed with water (first with tap water and second with distilled water), and their meat was cut into smaller portions (approximately 50–100 g). The meat portions were boiled at 100 °C for 30 min to separate water and oil. Then, the liquid was removed using a cloth strainer^35^, and they were stored at 70 °C for 24 h to eliminate any further moisture. After this, they were ground into powder using a meat grinder (commercial blender), sifted through a 500 μm mesh, and kept in a sterile place^23^. The yield was 182 g of biocoagulant (devilfish meal) per kg wet weight devilfish meat.

The thermal decomposition of the devilfish meal was studied by a thermogravimetric analysis technique (TA Instruments TGA Q500). Approximately 18.94 mg of sample was analyzed in a nitrogen atmosphere and a range of temperatures from ambient to 600 °C. A heating rate of 5 °C/min was used. Likewise, the devilfish meal’s cadmium, mercury, and lead concentrations were analyzed by Inductively Coupled Plasm-Mass Spectrometry (ICP-MS), determined in triplicate. These results were compared with the maximum limits of the heavy metals content in fishery products stipulated by the Health Official Mexican Standards NOM-242-SSA1-2009^36^.

### Experimental design

An experimental design was performed to evaluate the effectiveness of the biocoagulant. A combined design of Mixture-Process was selected. This design consisted of a mixture of three components (biocoagulant, aluminum sulfate, and ferric sulfate) and a numeric factor (coagulant doses, which ranged from 200 to 800 mg/L). Biocoagulant doses ranges have been reported between 1 and 120 mg /L^31^, and between 100 and 1000 mg/L^30^. Hence, it was decided to study the effect of the dose in a wider range taking values from 200 to 800 mg/L. The design resulted in 20 trials (Table 1) performed at the laboratory in random order. The component values were interpreted as proportions of the coagulant doses to be used. The proportion levels for each component ranged from 0 to 1, and the sum for each trial of the mixture was 1. A combined cubic x linear model with five replicates using a point exchange was selected. The removal percentages of turbidity, total suspended solids (TSS), and COD were the three responses to be observed and analyzed.

**Table 1:** Trials number obtained through experimental design.

Trials number	Doses (mg/L)	Components (proportion)		
		Biocoagulant	Aluminum sulfate	Ferric sulfate
1	200.00	0.667	0.000	0.333
2	800.00	0.667	0.000	0.333
3	200.00	0.000	0.667	0.333
4	800.00	0.000	0.667	0.333
5	200.00	0.333	0.333	0.333
6	200.00	0.333	0.667	0.000
7	200.00	0.333	0.000	0.667
8	800.00	0.000	0.333	0.667
9	200.00	0.000	0.333	0.667
10	200.00	0.000	1.000	0.000
11	200.00	0.000	0.000	1.000
12	200.00	0.667	0.333	0.000
13	200.00	1.000	0.000	0.000
14	800.00	0.000	1.000	0.000
15	800.00	0.333	0.667	0.000
16	500.00	0.500	0.500	0.000
17	500.00	0.000	0.000	1.000
18	200.00	0.000	0.667	0.333
19	800.00	0.000	0.667	0.333
20	200.00	0.333	0.333	0.333

### Jar test

The wastewater was mixed well before making the jar test, and the experiments were performed at the ambient temperature. Beakers were filled with 500 mL of ceramic industry wastewater and placed onto the floc illuminator. The jar test was performed using a jar floc test equipment (Flocculator SW6, Bibby Stuart, Armfield). Each test involved three steps: (1) rapid mixing at 150 rpm for 5 min with coagulants addition according to the experimental design (Table 1), (2) slow mixing at 50 rpm for 30 min with flocculant addition of 0.5 mL, and (3) sedimentation for 60 min. After the sedimentation, a volume of liquor supernatant (~ 50 mL) was pulled at 5 cm from the sample surface for conducting the water physicochemical characterization. Each test was performed in duplicate, and the average value was reported.

### Water physicochemical characterization

Before the jar test, the wastewater samples were physiochemically characterized by analyzing temperature, electrical conductivity, pH, turbidity, COD, and TSS. Temperature, electrical conductivity, pH, and turbidity were measured using a Mercury thermometer, a conductivity meter with RS-232 Cable (Oakton), a LAQUAact PH110 Potentiometer (Horiba Scientific), and a TB200TM Portable Turbidimeter (Orbeco-Hellige), respectively. COD and TSS were determined using the methods of the Mexican Standards NMX-AA-030/2-SCFI-2011^37^ and NMX-AA-034-SCFI-2015^38^, respectively.

After the jar test, turbidity, COD, and TSS were measured to calculate the removal percentage. The removal percentages of turbidity, COD, and TSS were used as the responses in the experimental design, being calculated by Eq. (1).

1 $${\text{Removal }}\left( \% \right) = 100 \times \left( {{\text{X}}_{{{\text{initial}}}} - {\text{X}}_{{{\text{final}}}} } \right)/{\text{X}}_{{{\text{initial}}}}$$

X_initial_ and X_final_ were the turbidity, COD, and TSS values before and after the coagulation process, respectively. The results were expressed as the mean of two measurements.

Furthermore, the pH was analyzed to identify a possible variation of this parameter when the jar test is performed.

### Statistical analysis and optimization

An analysis of variance ANOVA was performed for each response (turbidity, COD, and TSS) using Design-Expert® Version 12 Software (Trial version). The significance of the model and the lack of fit were established at a *p* value < 0.05. 3D surface plots and final model equations in terms of the mixture’s components and the numeric factor were presented by response type. Finally, an optimization analysis was performed under the conditions of the mixture’s components (range 0–1) and the doses (range 200–800 mg/L) to obtain the highest removal percentage of the three quality parameters using the obtained final model equations.

## Results and discussion

### TGA, and heavy metals content for devilfish meal (biocoagulant)

**Figure 1:**
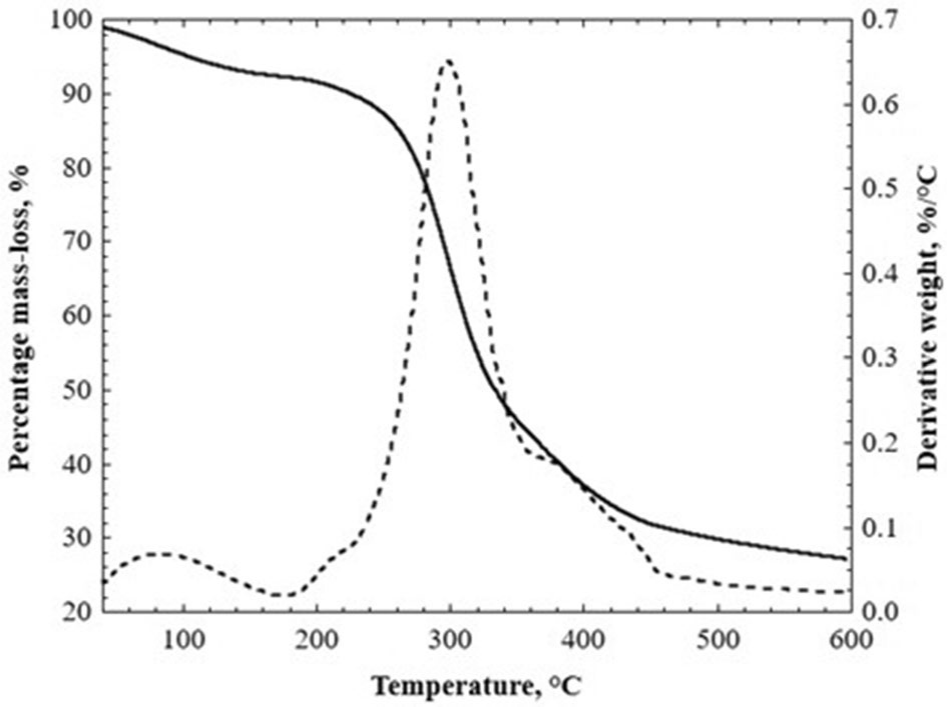
TGA thermogram for devilfish meal.

TGA is a technique used to determine the composition of a material and predict its thermal stability at temperatures up to 1000 °C. It measures the amount and change rate of the mass loss of a material as a function of temperature or time in a controlled atmosphere^39^. Figure 1 shows the thermal degradation curve’s TGA thermogram (solid line) and its first derivative (dotted line) for devilfish meal, where three different regions related to the most pronounced mass losses are observed. The principal mass loss is presented in three different thermal events with temperature ranges given by 40–175 °C, 175–350 °C, and 350–600 °C, respectively.

The first thermal event (40–175 °C) corresponds to the loss of the physisorbed and chemical water in the devilfish meal^40^, representing a mass loss of 7.7%. The most representative weight loss (49.4%) occurred in the second thermal event (175–350 °C). At this point, carbohydrates, and high molecular weight polysaccharides such as proteins, lipids, and other organic compounds are breaking down since this weight loss is in the range of temperatures where the degradation of starch and protein occurs^39^. These losses are also related to the combustion of collagen^40^, which is the interest component since it was thought to take advantage of this property so that the devilfish meal could act as a biocoagulant. Finally, the devilfish meal had a weight loss of 42.9% in the third thermal event (350–600 °C). This loss indicates that devilfish meal contains minerals and coupled salts such as calcium and phosphorus compounds^39,40^. The total mass loss of the devilfish meal was obtained at 600 °C.

The concentrations of cadmium, mercury, and lead of the devilfish meal were determined by ICP-MS. The results were 0.003 ± 0.01 mg/kg, 0.051 ± 0.04 mg/kg, and 1.211 ± 0.08 mg/kg, respectively. The cadmium and mercury concentrations are within the reference value (0.5 mg/kg) stipulated by the Health Official Mexican Standards NOM-242-SSA1-2009^36^. However, the lead concentration is 1.2 times higher than the reference value (1.0 mg/kg) established by the mentioned standard^36^. Although some authors have proposed its use as a source of proteins of high commercial value in food production and as a replacement for commercial fish meal^22–24^. Other authors suggest that the consumption of devilfish meat by humans and the use of devilfish meal as feed for fishes must be evaluated due to the possible health risk by the exposition to heavy metals bioaccumulated in these species^16^, such is the case of the lead content found in the devilfish meal produced in this work.

One of the reasons for the high lead content found in the devilfish meal could be the contamination that affects the water quality. Agriculture, mining, and food processing are the main industrial activities in Mexico, and their wastewater may increase the level of coastal contamination to be discharged without previous treatment. Some studies have reported high cadmium and lead concentrations in commercially important aquatic organisms of the coastal zone of Mexico^41,42^. If devilfish has contact with this contaminated water, it is exposed to the bioaccumulation of heavy metals in its body. On the other hand, devilfish is characterized by being a predatory species^17^, and some authors indicate that especially predatory marine species bioaccumulate heavy metals over a long lifetime^43^. So, due to the possibility of representing health risks of the consumption of devilfish meat and the consumers’ resistance to integrating it into their diet, an alternative is its use to produce a biocoagulant since its lead content does not represent a health risk during wastewater treatment. The World Health Organization (WHO) establishes a quality reference value of 0.01 mg/L of lead in drinking water^44^, and assuming that the biocoagulant is used at the maximum concentration proposed in this work (800 mg/L), a lead concentration of 0.0001 mg/L would be introduced, being a value 110 times lower than the reference value.

### Water physicochemical characterization before coagulation treatment

Ceramic tile effluent contains significant concentrations of fine suspended particles of clay minerals and dyestuffs or glazes, resulting in colored wastewater and high concentrations of turbidity and suspended solids^45^. Such effluents may severely negatively impact the receiving water quality when discharged untreated. Physicochemical characterization of the samples of ceramic industry wastewater before the coagulation treatment is shown in Table 2.

**Table 2:** Ceramic industry wastewater physicochemical characterization.

Parameters	Value*
Temperature (°C)	8.00 ± 1.00
Electrical conductivity (µS/cm)	543 ± 0.00
pH	8.17 ± 0.02
Turbidity (NTU)	> 1000 ± 0.00
COD (mg/L)	318.14 ± 2.74
TSS (mg/L)	0.80 ± 0.02

*The value shows the mean of three measures.

The temperature of ceramic industry wastewater (Table 2) complies with the recommended limit (< 25 °C) by the WHO since microorganisms can proliferate at temperatures above 25 °C and may increase problems related to odor, color, taste, and corrosion^44^. The electrical conductivity indicates the salt content or dissolved ionic components in water^46^. Studies of freshwaters suggest that streams supporting well-mixed fisheries must range between 150 and 500 µS/cm. Outside this range could indicate that the water is unsuitable for certain aquatic organisms^47^. The value found in the ceramic industry wastewater is close to or slightly above the range (Table 2). Therefore, it could cause an environmental risk to direct discharge in water bodies. The pH of the ceramic industry wastewater is alkaline (Table 2) and is within the quality range (6.5–8.5) established by the WHO for any purpose^44^.

The WHO establishes a quality value for turbidity in water < 5 NTU^44^. The turbidity value obtained in this study (Table 2) exceeded ~ 200 times more than the quality parameter. Excessive turbidity in water may increase treatment costs due to problems caused in the flocculation, filtration, and disinfection processes. Likewise, it also makes the sight of the receiving water bodies, where the effluent is being discharged, unpleasant for full-contact recreation^48^. Therefore, ceramic industry wastewater requires a treatment based on coagulation-flocculation processes since it would be difficult to remove the turbidity at parameters < 5 NTU using only a sedimentation process.

The COD measures the susceptibility to oxidation of the organic and inorganic materials present in water bodies and effluents from wastewater treatment plants. The Mexican water quality guidelines specify COD limit concentrations for the wastewater discharge between 60 and 210 mg/L^49^. The United States Environmental Protection Agency and the German Environment Agency established COD discharge limits of 125 mg/L and 150 mg/L for urban wastewater treatment plants^50^. The COD value of the ceramic industry wastewater (Table 2) is 1.5 times higher than the mentioned upper limit, so it would be expected that the COD-causing substances would be removed by coagulation treatment.

Water quality guidelines for suspended solids concentrations in freshwater systems have been suggested. Although, some authors note that the reference values are based on the concept of turbidity, being a surrogate measure of the concentration of solids. Hence, they are not often valid^51^. Canadian Council of Ministers of the Environment (CCME) suggests that suspended solids concentrations should not increase by more than 25 mg/L from background levels for any short-term exposure (maximum 1 day) and more than 5 mg/L from background levels for any long-term exposure (between 1 and 13 days)^52^. The TSS value of the ceramic industry wastewater (Table 2) is within both mentioned limits. Therefore, this parameter does not cause a water quality deterioration. However, it was considered necessary due to its relationship with turbidity. Some authors have reported that turbidity is influenced by the shape and particle size of suspended solids, the presence of phytoplankton, and dissolved humic and mineral substances. Hence, a high turbidity reading can be recorded without requiring a high TSS concentration^51^. This condition could be an issue of the low values obtained in this study, linked to the fact that very fine particles are responsible for turbidity and were not retained during the filtration.

### Water physicochemical characterization after coagulation treatment

The physicochemical characterization of the samples of ceramic industry wastewater after the coagulation treatment is shown in Table 3.

**Table 3:** Ceramic industry water physicochemical characterization after the coagulation treatment.

Trials number	Doses (mg/L)	ΔpH (pH_final_ − pH_initial_)	Removal		
			Turbidity (%)	COD (%)	TSS (%)
1	200.00	− 0.4	68.9	76.0	98.0
2	800.00	− 1.3	48.6	68.8	86.0
3	200.00	− 1.3	68.6	68.3	66.0
4	800.00	− 2.0	89.7	88.9	100.0
5	200.00	− 1.1	55.7	63.2	90.0
6	200.00	− 1.0	64.6	65.4	90.0
7	200.00	− 0.9	73.4	73.9	84.0
8	800.00	− 2.4	76.8	88.8	84.0
9	200.00	− 1.1	19.0	48.2	68.0
10	200.00	− 1.6	27.8	51.2	100.0
11	200.00	− 1.2	63.7	65.1	82.0
12	200.00	− 0.3	68.0	58.1	54.0
13	200.00	− 0.3	67.4	56.9	50.0
14	800.00	− 2.3	42.8	69.3	98.0
15	800.00	− 1.5	50.7	72.4	100.0
16	500.00	− 0.7	62.2	66.7	90.0
17	500.00	− 2.0	74.0	81.4	64.0
18	200.00	− 1.6	80.9	81.3	60.0
19	800.00	− 2.0	31.9	87.4	96.0
20	200.00	− 1.1	66.3	63.0	66.0

Data recorded in Table 3 revealed that the pH of the wastewater samples slightly changed after the coagulation treatment. The more remarkable changes (< − 1.5) in pH occur when only ferric sulfate or aluminum sulfate is used or in the mixture of both chemical coagulants. These results indicate that the biocoagulant is not affecting pH values during the wastewater treatment. Chemical coagulation is a process highly dependent on pH changes. Therefore, the specific operational pH needs to be analyzed for each coagulant type^53^. The optimal pH range for ferric salts is > 5, and aluminum salts range from 6.5 to 7.2^54^. The pH of the wastewater samples was not adjusted to the optimal condition of each coagulant used since the intention was to test the effect of the biocoagulant and the chemical agents without adjusting the pH of the wastewater samples. Some authors have reported that the biocoagulants have the advantage of not being sensitive to pH^55^. This action would facilitate an escalation of the treatment without acidifying or basifying the water to treat. However, the ceramic industry wastewater with a pH above 8 (Table 2) could affect the performance of the chemical agents (ferric sulfate and aluminum sulfate) used in the jar tests, and these chemical agents could acidify the pH to a value out of the recommended limit to discharge treated water.

The removal percentages of turbidity, COD, and TSS in Table 3 were taken as the responses in the experimental design. Their analysis and discussion are presented in “ANOVA results and 3D response surface plots”.

### ANOVA results and 3D response surface plots

Table 4 shows the ANOVA results obtained for each water quality parameter (turbidity, COD, and TSS) and the final equations in terms of components and the factor (doses) by response type. It can be noted that the models are statistically significant (*p* value ≤ 0.05), and the lack of fit is not statistically significant (*p* value ≥ 0.05), so the equations are adequate to describe the observed data. These data were fitted to a combined reduced special cubic x linear model. The special cubic model includes the linear part, double and triple interactions, which are referred to the mixture’s proportions as variables, and the linear model includes the effect of the used doses as a numeric factor. The model was reduced to terms that are only significant. Hence, it can be observed that the significant effects were the linear mixture of the three components (coagulants) and some double interactions between the coagulants and the doses for the case of the turbidity as the response. The linear mixture of the three components (coagulants) and some double and triple interactions between the coagulants and the doses were the significant effects for COD and TSS responses. The analysis of variance ANOVA in detail for turbidity, COD, and TSS are shown in Tables S1, S2, and S3 (Supplementary materials), respectively.

**Table 4:** ANOVA results and final equations in terms of components and factor by response type.

Water quality parameter	Model significance (*p* value)	Lack of fit significance (*p* value)	Equations
Turbidity	< 0.0001	0.8144	Turbidity = 64.50 × A + 48.02 × B + 48.46 × C + 27.01 × A × B + 0.04 × C × D
COD	< 0.0001	0.0886	COD = 56.49 × A + 60.36 × B + 64.95 × C − 13.84 × A × B + 49.91 × A × C − 2.83 × 10^−4^ × A × D − 1.71 × B × C + 6.27 × 10^−3^ × B × D + 0.01 × C × D − 207.46 × A × B × C + 0.09 × A × B × D + 0.10 × B × C × D
TSS	< 0.0001	0.4968	TSS = 47.39 × A + 97.79 × B + 72.55 × C − 0.72 × A × B + 133.02 × A × C + 1.77 × 10^−3^ × A × D − 112.50 × B × C + 1.44 × 10^−3^ × B × D − 3.35 × 10^−4^ × C × D + 0.09 × A × B × D + 0.15 × B × C × D

A: Biocoagulant, B: Aluminum sulfate, C: Ferric sulfate, D: Doses.

**Figure 2:**
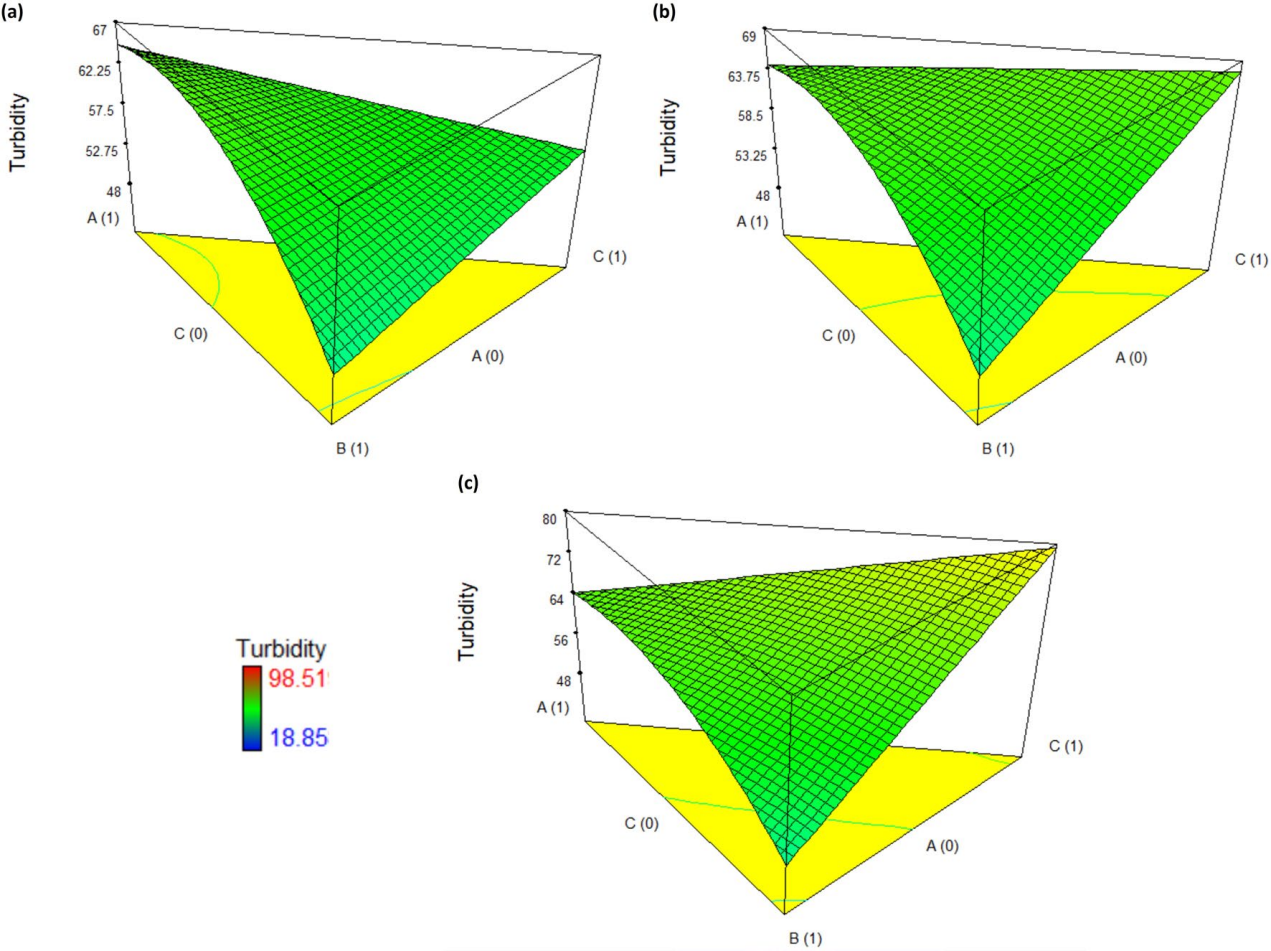
3D response surface plots for the turbidity removal in ceramic industry wastewater using coagulant doses: (**a**) low-200 mg/L, (**b**) medium-500 mg/L, and (**c**) high-800 mg/L.

**Figure 3:**
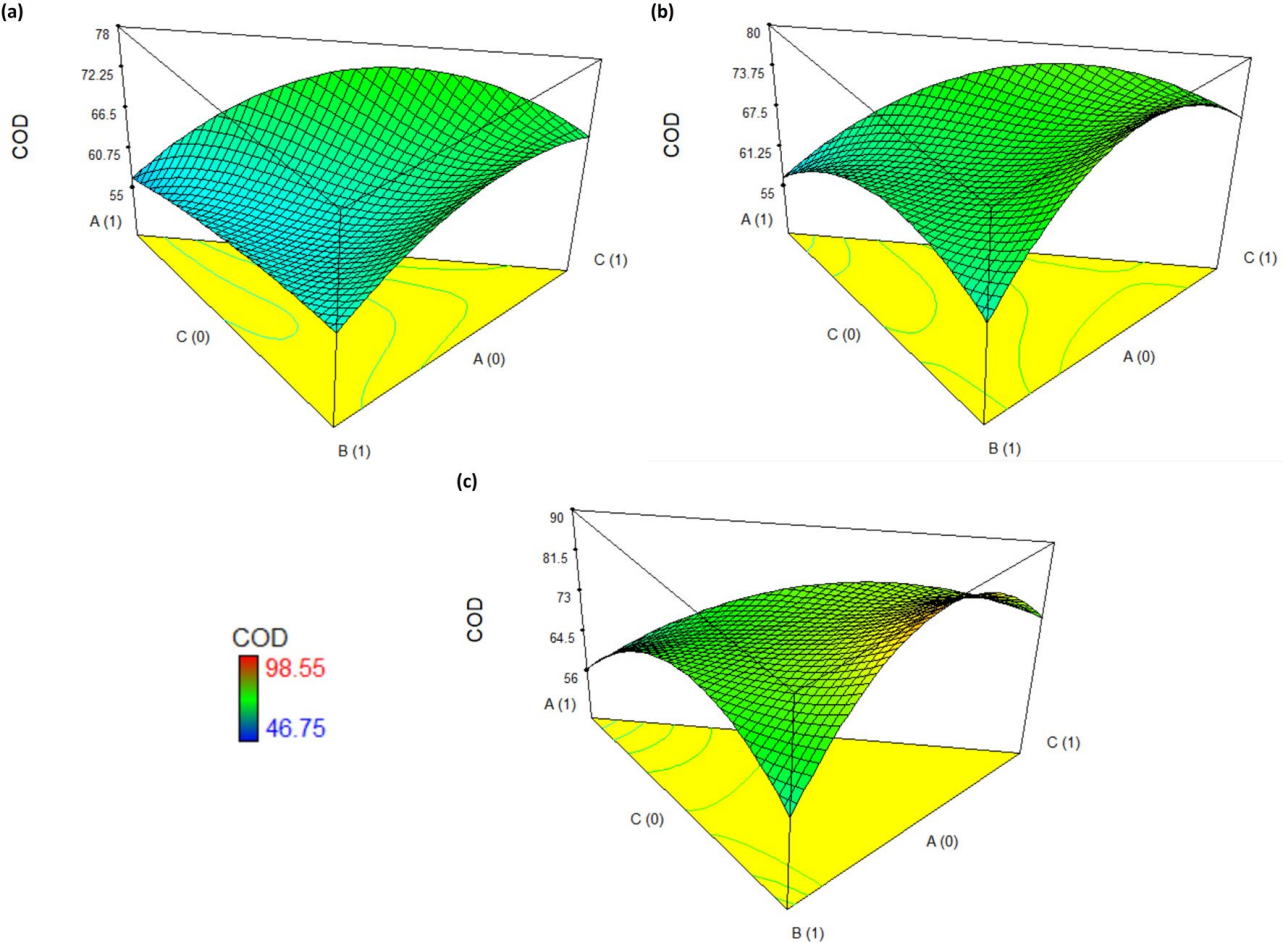
3D response surface plots for the COD removal in ceramic industry wastewater using coagulant doses: (**a**) low-200 mg/L, (**b**) medium-500 mg/L, and (**c**) high-800 mg/L.

**Figure 4:**
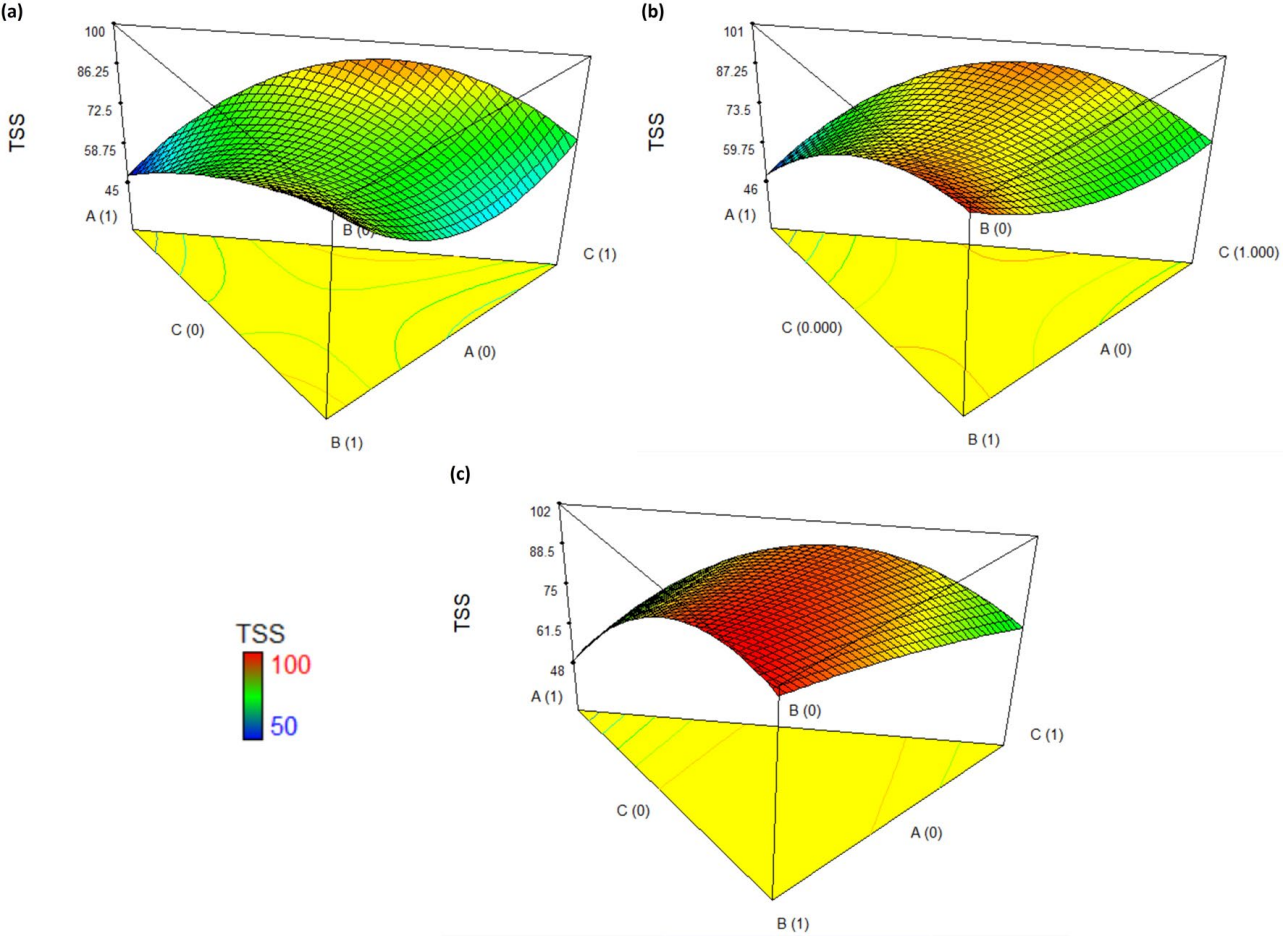
3D response surface plots for the TSS removal in ceramic industry wastewater using coagulant doses: (**a**) low-200 mg/L, (**b**) medium-500 mg/L, and (**c**) high-800 mg/L.

The 3D response surface plots for the removal of turbidity, COD, and TSS in ceramic industry wastewater are shown in Figs. 2, 3, and 4, respectively; being the responses analyzed for three doses: low (200 mg/L), medium (500 mg/L), and high (800 mg/L).

Considering the turbidity as a quality parameter, if the coagulation treatment was performed with a dose of 200 mg/L, a turbidity removal efficiency of 65.5% is achieved using a mixture of 80% biocoagulant and 20% aluminum sulfate (Fig. 2a). However, using a dose of 500 mg/L or 800 mg/L of ferric sulfate alone, efficiencies of 67.8% and 79.4% are achieved (Fig. 2b,c). Therefore, ferric sulfate has a more significant effect in removing turbidity from ceramic industry wastewater under conditions of doses higher than 500 mg/L. On the other hand, a mixture of 1:4 of aluminum sulfate and biocoagulant could significantly influence the turbidity removal, although not representing one of the best efficiencies (< 65%). Hence, an analysis of the effect on COD and TSS was also performed.

Regarding COD, if the coagulation treatment was performed using a dose of 200 and 500 mg/L, COD removal efficiencies of 75.1% and 77.4% are achieved with a mixture of 37% biocoagulant and 63% ferric sulfate (Fig. 3a,b). On the other hand, using a dose of 500 mg/L, achieving a COD removal efficiency of 79.5% with a mixture of 42% aluminum sulfate and 58% ferric sulfate is possible. Increasing the doses to 800 mg/L and maintaining the same mixture proportions of both sulfates (Fig. 3c), an efficiency approximated 89% is achieved. Furthermore, using a mixture of 31% biocoagulant and 69% ferric sulfate, an efficiency of 79.9% is achieved. So, a mixture of biocoagulant and ferric sulfate or both sulfates have a more significant effect in removing COD from ceramic industry wastewater.

Regarding TSS, if the coagulation treatment was performed using a dose of 200 mg/L, a TSS removal efficiency of 98.1% is achieved with aluminum sulfate alone (Fig. 4a). However, it is possible to achieve an efficiency of 94.5% with a mixture of 41% biocoagulant and 59% ferric sulfate. If the dose was increased to 500 mg/L using only aluminum sulfate, an efficiency of 98.5% is achieved. Likewise, with the same proportion of mixture of the biocoagulant (41%) and ferric sulfate (59%), a removal efficiency of 94.7% is obtained (Fig. 4b). Therefore, a dose increase would not be worthwhile when the improvement in efficiency is not so significant. These conditions vary with a coagulant dose of 800 mg/L, where a TSS removal of approximately 100% is achieved with a mixture of 22% biocoagulant and 78% aluminum sulfate or 41% biocoagulant and 59% ferric sulfate (Fig. 4c). Likewise, it is appreciated that increasing the dose of aluminum sulfate has more effect in removing TSS. However, ferric sulfate has a better effect, even when is mixed with the biocoagulant.

The optimal doses and the proportions of the mixture of the coagulants will depend on the interest in improving the water quality parameters. So, an optimization analysis was performed considering the maximum removal percentages obtained for the three quality parameters together. The obtained better condition was when the coagulation treatment was performed applying a dose of 800 mg/L with a mixture of 35% biocoagulant and 65% ferric sulfate, achieving removal efficiencies of 74.2%, 79.8%, and 94.3% for turbidity, COD, and TSS, respectively. Increasing the doses to 800 mg/L may not be feasible in terms of costs. However, it should be appreciated that reducing the dose could affect removal efficiencies. A trial was performed in the laboratory under these treatment conditions and with the same methodology, obtaining removal efficiencies of 73.1%, 77.2%, and 94.6% for turbidity, COD, and TSS, respectively. Therefore, an error of less than 3.3% is obtained in the model’s prediction.

### Discussion on the effectiveness of the biocoagulant

A biocoagulant can coagulant very fine particles and organic and inorganic matter suspended in water^55^ by promoting adsorption, polymer bridging, and charge neutralization mechanisms during the coagulation process^53^. Chitin and polysaccharides are the main characteristics of the shellfish to be used in coagulation processes^56^. The high protein content in devilfish meals (used as biocoagulant) could favor the formation of flocs since its effectiveness could be linked to the charge neutralization through the interaction of two particles with oppositely charged ions and to bridging mechanisms by the forming particle-polymer particle complexes during the adsorption of particles onto polymer chains. Other mechanisms such as electrostatic patch and sweeping, adsorption, complexation, chelation, entrapment, and precipitation may also contribute to the formation of flocs^56^. A comparison between the effectiveness of different biocoagulants applied to the water, and wastewater treatment with the results obtained in this study is shown in Table 5.

**Table 5:** Comparison of the effectiveness in water and wastewater treatment using biocoagulants.

Biocoagulant (source used)	Water type	Doses used	Effectiveness in the removal of contaminants	References
Devilfish meal	Ceramic industry wastewater	800 mg/L (mixture of 35% biocoagulant and 65% ferric sulfate)	74.2%, 79.8%, and 94.3% of turbidity, COD, and TSS, respectively	This study
*Jatropha curcas* seeds	Kaolin synthetic water	120 mg/L	> 96% of turbidity at pH 3	^31^
*Moringa oleifera*, *Dolichos lablab*, and *Cicer arietinum* seeds	Synthetic turbid water prepared by adding clay materials	100 mg/L	94.1, 88.9, and 95.89% of turbidity, respectively	^34^
*Cassia obtusifolia* seed gum	Palm oil mill effluent	1.15 mg/L alum, and 2.47 mg/L biocoagulant	81.6%, and 48.2% of TSS, and COD, respectively	^57^
*Ocimum basilicum* (basil)	Landfill leachate	High dose	64.4% of COD	^58^

Different biocoagulants have been used for industrial wastewater, natural and synthetic water treatment. Biocoagulants produced from seeds of *Jatropha curcas*, *Moringa oleifera*, and *Cicer arietinum* have efficiently removed the turbidity in water with clay and kaolin (Table 5). However, the biocoagulant produced from devilfish meal is more feasible in dual systems when mixed with ferric sulfate. Therefore, its application could represent an alternative for reducing the use of chemical coagulants, the decrease in sludge generation during the treatment, and the treatment cost as aid coagulant^59^. Based on Table 5, the use of aid coagulants as *Cassia obtusifolia* seed gum and *Ocimum basilicum* (basil) have reported improvements in removal efficiency of contaminants in different wastewater types^57,58^, such as the case of this study where the use of the biocoagulant from devilfish improves the removal efficiency of turbidity, COD, and TSS in ceramic industry wastewater.

The prices of biocoagulants must be competitive with those of chemical coagulants. The drinking water and wastewater treatment costs using biocoagulants range USD 0.0025-2 and USD 0.015-19.5 per cubic meter of treated water. In contrast, chemical coagulants’ water treatment costs are approximately USD 1.50 and USD 0.15-1.80, respectively^53^. Although some biocoagulants show a higher cost than using chemical coagulants, the cost could be reduced when the feedstock comes from waste, and the cost of the resultant sludge handling is also included.

The aluminum coagulants have the disadvantage of producing a less dense floc than iron coagulants. However, iron coagulants have the disadvantage of increasing up 40% the weight of sludge compared to aluminum coagulants^54^. A high dose (800 mg/L) of ferric sulfate is required to remove efficiently turbidity, COD, and TSS of the ceramic industry wastewater. Hence, one aspect that must be considered is the generation of more sludge. However, when the ferric sulfate is mixed with the biocoagulant, the amount of sludge could be reduced. The quantification of sludge generated was not estimated during the jar tests, so it is suggested to be evaluated in the future. Furthermore, applying the coagulation treatment in the ceramic industry wastewater requires adequate disposal of the generated sludge in landfills. Although, this waste could also be valorized as fill material in the construction or as raw material in the sanitary ware production^60^.

Yonge^61^ reported USD 0.20–0.30/kg costs for ferric sulfate coagulant. Salas-Razo et al.^62^ estimated a supply market to the price of USD 0.07/kg of devilfish and a cost of production of silage acid of devilfish in USD 0.14/kg. This last price could be associated with the production cost of devilfish meals. Although the production cost of the produced biocoagulant was not calculated in this work, and it could be subjective, the values shown reflect that the biocoagulant from devilfish meal could represent a lower-cost alternative material than conventional coagulants.

Finally, applying dual systems reduce the treatment cost and the use of chemical coagulants. Based on the optimization results obtained in this study, the treatment cost in the coagulation process could reduce when 280 mg/L of biocoagulant and 520 mg/L of ferric sulfate instead of 800 mg/L of ferric sulfate are used in the ceramic industry wastewater. Hence, the biocoagulant from devilfish could represent a biodegradable and low-cost material in wastewater treatment.

## Conclusions

This study produced a biocoagulant from devilfish meal, an invasive species that cause socioeconomic and ecological impacts, and its meal could have heavy metals putting human health at risk if was consumed. The effectiveness of the biocoagulant and two chemical coagulants (ferric sulfate and aluminum sulfate) was evaluated for the removal of turbidity, COD, and TSS from ceramic industry wastewater using a combined experimental design of Mixture-Process. This design optimized the operation conditions of the coagulation processes found that removal efficiencies of 74%, 79%, and 94% for turbidity, COD, and TSS, respectively, are achieved using an optimal coagulant dose of 800 mg/L with a mixture of 35% biocoagulant and 65% ferric sulfate. ANOVA results showed that the models are significant (*p* value < 0.0001), and the lack of fit is not required (*p* value > 0.05). Hence, the experimental data were fitted to a combined reduced special cubic x linear model, having the model prediction an error of 3.3% concerning observed results. These results support the use of devilfish meal as a biocoagulant, being more feasible in dual systems when mixed with ferric sulfate.

## Supplementary Information

Supplementary Information.

## Data Availability

The datasets generated and/or analyzed during the current study are available from the corresponding author on reasonable request.
