# Supplementary material for: Evaluation of a biocoagulant from devilfish invasive species for the removal of contaminants in ceramic industry wastewater
Source: Sci Rep. 2022 Jun 15;12:9917. doi: 10.1038/s41598-022-14242-6 (PMC9200807; doi:10.1038/s41598-022-14242-6)
Supplement: Supplementary file 1 — Supplementary Information. [file 41598_2022_14242_MOESM1_ESM.docx]

SUPPLEMENTARY MATERIALS_TABLES

**Evaluation of a biocoagulant from devilfish invasive species for the removal of contaminants in ceramic industry wastewater**

Miguel Mauricio Aguilera Flores, Nahum Andrés Medellín Castillo, Verónica Ávila Vázquez, Raúl González García, Antonio Cardona Benavides, and Candy Carranza Álvarez

**Table S1**. Analysis of variance for turbidity.

| **Source** | **Sum of squares** | **Degree of freedom** | **Mean Square** | **F Value** | **p-value** | **Remark** |
| --- | --- | --- | --- | --- | --- | --- |
| Model | 13444.97 | 7 | 1920.71 | 7.69 | < 0.0001 | Significant |
| Linear Mixture | 2568.17 | 2 | 1284.08 | 5.14 | 0.0116 |  |
| AB | 1601.17 | 1 | 1601.17 | 6.41 | 0.0165 |  |
| AE | 2048.79 | 1 | 2048.79 | 8.20 | 0.0073 |  |
| BE | 4782.64 | 1 | 4782.64 | 19.14 | 0.0001 |  |
| CD | 931.84 | 1 | 931.84 | 3.73 | 0.0624 |  |
| ABE | 747.99 | 1 | 747.99 | 2.99 | 0.0932 |  |
| Residual | 7995.92 | 32 | 249.87 |  |  |  |
| Lack of fit | 6140.64 | 27 | 227.43 | 0.61 | 0.8144 | Not significant |
| Pure Error | 1855.28 | 5 | 371.06 |  |  |  |
| Cor Total | 21440.90 | 39 |  |  |  |  |

A: Biocoagulant, B: Aluminum sulfate, C: Ferric sulfate, D: Doses, E: Wastewater type.

**Table S2**. Analysis of variance for COD.

| **Source** | **Sum of squares** | **Degree of freedom** | **Mean Square** | **F Value** | **p-value** | **Remark** |
| --- | --- | --- | --- | --- | --- | --- |
| Model | 7719.74 | 16 | 482.48 | 7.77 | < 0.0001 | Significant |
| Linear Mixture | 2122.56 | 2 | 1061.28 | 17.09 | < 0.0001 |  |
| AB | 876.60 | 1 | 876.60 | 14.12 | 0.0010 |  |
| AC | 198.28 | 1 | 198.28 | 3.19 | 0.0871 |  |
| AD | 0.025 | 1 | 0.025 | 0.0004 | 0.9841 |  |
| AE | 44.36 | 1 | 44.36 | 0.71 | 0.4067 |  |
| BC | 451.18 | 1 | 451.18 | 7.27 | 0.0129 |  |
| BD | 15.64 | 1 | 15.64 | 0.25 | 0.6205 |  |
| BE | 1736.14 | 1 | 1736.14 | 27.96 | < 0.0001 |  |
| CD | 52.17 | 1 | 52.17 | 0.84 | 0.3688 |  |
| CE | 7.91 | 1 | 7.91 | 0.13 | 0.7244 |  |
| ABC | 180.30 | 1 | 180.30 | 2.90 | 0.1018 |  |
| ABD | 133.48 | 1 | 133.48 | 2.15 | 0.1561 |  |
| ABE | 352.19 | 1 | 352.19 | 5.67 | 0.0259 |  |
| ACE | 44.25 | 1 | 44.25 | 0.71 | 0.4072 |  |
| BCD | 187.13 | 1 | 187.13 | 3.01 | 0.0959 |  |
| Residual | 1427.97 | 23 | 62.09 |  |  |  |
| Lack of fit | 1320.99 | 18 | 73.39 | 3.43 | 0.0886 | Not significant |
| Pure Error | 106.98 | 5 | 21.40 |  |  |  |
| Cor Total | 9147.71 | 39 |  |  |  |  |

A: Biocoagulant, B: Aluminum sulfate, C: Ferric sulfate, D: Doses, E: Wastewater type.

**Table S3**: Analysis of variance for TSS.

| **Source** | **Sum of squares** | **Degree of freedom** | **Mean Square** | **F Value** | **p-value** | **Remark** |
| --- | --- | --- | --- | --- | --- | --- |
| Model | 6460.32 | 15 | 430.69 | 6.15 | < 0.0001 | Significant |
| Linear Mixture | 1765.05 | 2 | 882.52 | 12.61 | 0.0002 |  |
| AB | 432.51 | 1 | 432.51 | 6.18 | 0.0203 |  |
| AC | 789.09 | 1 | 789.09 | 11.28 | 0.0026 |  |
| AD | 1.00 | 1 | 1.0 | 0.014 | 0.9059 |  |
| AE | 516.28 | 1 | 516.28 | 7.38 | 0.0121 |  |
| BC | 45.98 | 1 | 45.98 | 0.66 | 0.4256 |  |
| BD | 0.83 | 1 | 0.83 | 0.012 | 0.9142 |  |
| BE | 21.94 | 1 | 21.94 | 0.31 | 0.5807 |  |
| CD | 0.037 | 1 | 0.037 | 0.0005 | 0.9819 |  |
| CE | 340.12 | 1 | 340.12 | 4.86 | 0.0373 |  |
| ABD | 145.97 | 1 | 145.97 | 2.09 | 0.1616 |  |
| ACE | 946.27 | 1 | 946.27 | 13.52 | 0.0012 |  |
| BCD | 442.30 | 1 | 442.30 | 6.32 | 0.0190 |  |
| BCE | 129.67 | 1 | 129.67 | 1.85 | 0.1861 |  |
| Residual | 1679.57 | 24 | 69.98 |  |  |  |
| Lack of fit | 1358.92 | 19 | 71.52 | 1.12 | 0.4968 | Not significant |
| Pure Error | 320.64 | 5 | 64.13 |  |  |  |
| Cor Total | 8139.88 | 39 |  |  |  |  |

A: Biocoagulant, B: Aluminum sulfate, C: Ferric sulfate, D: Doses, E: Wastewater type.
